# Absence of GDF15 Aggravates Pressure Overload-Induced Cardiac Remodelling in Mice Hallmarked by Perivascular Fibrosis and Signs of Endothelial-to-Mesenchymal Transition

**DOI:** 10.3390/ijms27125387

**Published:** 2026-06-15

**Authors:** Marian Wesseling, Gonzalo Sanchez-Duffhues, Judith J. de Haan, Jasper Tromp, Lena Bosch, J. Conny van Munsteren, Maike A. D. Brans, Joost P. G. Sluijter, Gerard Pasterkamp, Marie-José Goumans, Saskia C. A. de Jager

**Affiliations:** 1 Laboratory of Experimental Cardiology, University Medical Centre Utrecht, 3584 CX Utrecht, The Netherlandsl.bosch@umcutrecht.nl (L.B.); m.a.d.brans@umcutrecht.nl (M.A.D.B.); j.sluijter@umcutrecht.nl (J.P.G.S.); 2 Central Diagnostic Laboratory, University Medical Centre Utrecht, 3584 CX Utrecht, The Netherlands; g.pasterkamp@umcutrecht.nl; 3 Department of Cell and Chemical Biology, Leiden University Medical Centre, 2300 RC Leiden, The Netherlands; g.s.duffhues@cinn.es; 4 Nanomaterials and Nanotechnology Research Center (CINN-CSIC), Health Research Institute of Asturias (ISPA), 33011 Oviedo, Asturias, Spain; 5 Saw Swee Hock School of Public Health, National University of Singapore and the National University Health System, Singapore 119228, Singapore; jasper_tromp@nus.edu.sg; 6 Duke-NUS Medical School, Singapore 169857, Singapore; 7 National Heart Centre Singapore, Singapore 169609, Singapore; 8 Department of Anatomy and Embryology, Leiden University Medical Centre, 2300 RC Leiden, The Netherlands; j.c.van_munsteren@lumc.nl; 9 UMC Utrecht Regenerative Medicine Centre, Circulatory Health Laboratory, 3584 CX Utrecht, The Netherlands; 10 Facultity of Medical Sciences, Utrecht University, 3584 CS Utrecht, The Netherlands

**Keywords:** growth differentiation factor-15, cardiac function, heart failure, EndMT, endothelial dysfunction, fibrosis, TGF-β

## Abstract

Growth differentiation factor 15 (GDF15) levels are associated with increased mortality and rehospitalisation in heart failure (HF) patients. Whether GDF15 is causally involved in the pathobiology of HF remains largely unknown. Using the transverse aortic constriction (TAC) mouse model, we investigated the role of GDF15 in pressure overload-induced HF. Following TAC, circulating GDF15 levels increased significantly. Compared to wild type (WT) littermates, genetically deficient *Gdf15*^-/-^ mice developed more pronounced adverse cardiac remodelling one week after TAC, characterised by increased cardiac volumes and impaired myocardial global deformation. This further aggravated into severe HF in *Gdf15*^-/-^ mice over 42 days follow-up. Cardiac remodelling in *Gdf15*^-/-^ was accompanied by enhanced perivascular fibrosis and increased co-localization of fibroblast- and endothelial-specific markers in the cardiac endothelium of *Gdf15*^-/-^ mice, suggestive of endothelial plasticity and Endothelial-to-Mesenchymal transition (EndMT)-like changes. To further explore potential endothelial mechanisms underlying these observations, we performed complementary in vitro experiments in GDF15 knockdown endothelial cells. GDF15 deficiency impaired barrier function and enhanced Activin A-induced mesenchymal marker expression, consistent with increased endothelial phenotypic modulation. Together, these findings demonstrate that the loss of GDF15 aggravates pressure overload-induced heart failure, hallmarked by perivascular fibrosis and signs of endothelial dysfunction. Our data further support a potential protective role for GDF15 in maintaining endothelial integrity during cardiac stress.

## 1. Introduction

During the last two decades, the prevalence of heart failure (HF) has increased significantly [1,2]. Though treatment options for HF patients have improved, there is a remaining and urgent need to better understand the pathology to improve early diagnosis and treatment. High circulating growth differentiation factor 15 (GDF15) levels have been associated with poor prognosis in various cardiovascular diseases, ranging from acute coronary syndromes to established HF [3,4,5,6,7,8]. The growth factor GDF15 is a distant member of the transforming growth factor (TGF)-β family [9], which includes approximately 30 members subclassified into different homology groups that include GDFs, TGF-βs, Activins, and bone morphogenetic proteins (BMPs). Notably, disturbances in the TGF-β balance, including gene mutations, are often associated with an increased risk of cardiovascular disease [10,11]. High GDF15 levels constitute a risk factor for worse outcomes and death in the development of adverse left ventricular remodelling and HF [12,13,14,15,16]. Several studies have examined the potential benefits of GDF15 for risk stratification and prognostication in patients with HF [14,17,18,19,20,21]. Moreover, GDF15 provides prognostic information on the risk of death beyond that of established clinical biomarkers [7], which suggests the benefit of including GDF15 as a component of a multi-biomarker study [1,14,17,18,22].

Several studies describe the wide range of cellular processes involving GDF15, including inflammation, apoptosis, matrix remodelling, and cardiomyocyte hypertrophy [23]. Xu and colleagues have shown that overexpression of GDF15 protects, while GDF15 knockdown aggravates early adverse cardiac remodelling upon TAC, associating these effects with cardiomyocyte hypertrophy [24]. In addition, it was shown that GDF15 plays an important role as a mediator in inflammation-driven tissue tolerance to damage [25]. Indeed, GDF15 prevents cardiac damage and failure by promoting cellular survival during the acute phase of inflammation [25].

Disturbed vascular function is a common contributor to cardiovascular disease, including HF [26,27]. Particularly, in HF with preserved ejection fraction (HFpEF), it has been postulated that a systemic pro-inflammatory state induces microvascular endothelial dysfunction resulting in excessive perivascular fibrosis leading to stiffening of the myocardium [28,29]. Although TGF-β signalling is recognised as a key contributor to cardiac fibrosis and remodelling, how GDF15 balances the physiological functions of the TGF-β family in cardiovascular disease remains largely unaddressed. The induction of Endothelial-to-Mesenchymal Transition (EndMT) by members of the TGF-β family has been identified as an extreme form of endothelial plasticity that contributes to the onset and progression of perivascular cardiac fibrosis [30]. In addition, an increased cardiac inflammatory response accelerates endothelial dysfunction, thereby directly compromising microvessel stability and favouring HF progression by high diastolic left ventricular stiffness, ultimately increasing mortality [26,28].

There is accumulating evidence suggesting that GDF15 is an important determinant in fibrotic response mechanisms following tissue injury. However, whether GDF15 is directly involved in the pathogenesis of HF or merely reflects the degree of cardiac damage and severity of HF remains largely unknown. In this study, we investigated the role of GDF15 in HF of non-ischemic origin using in vivo and in vitro disease models. We show that GDF15 deficiency induced vascular dysfunction, including EndMT-like endothelial changes, leading to perivascular fibrosis, cardiac hypertrophy, and severe HF.

## 2. Results

### 2.1. Circulating GDF15 Levels Increase upon Pressure-Overloaded Heart Failure in Mice

**Figure 1:**
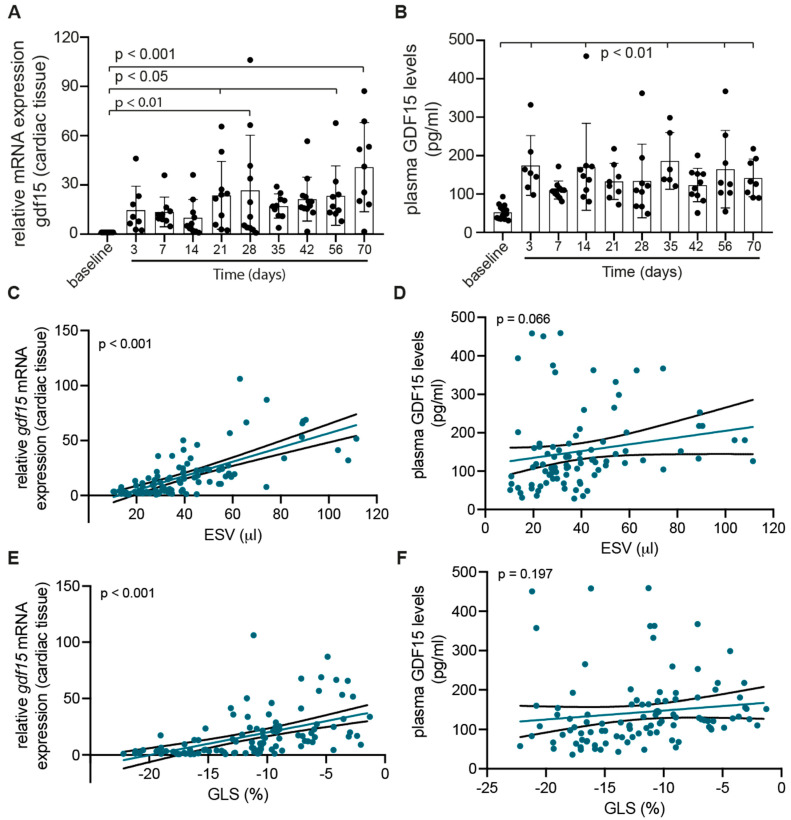
Circulating GDF15 levels increased with heart failure induced by pressure overload. Upon induction of pressure overload heart failure, mRNA *gdf15* expression in the heart (**A**) and plasma GDF15 levels (**B**) significantly increase compared to baseline. *Gdf15* mRNA expression n = 13 at baseline, n = 8 at 3, n = 10 at 7, n = 7 at 14, n = 9 at 21, n = 10 at 28, n = 9 at 35, n = 9 at 42, n = 8 at 56, and n = 9 at 70 days follow-up; plasma GDF15 levels n = 12 at baseline, n = 8 at 3, n = 10 at 7, n = 7 at 14, n = 19 at 21, n = 9 at 28, n = 9 at 35, n = 8 at 42, n = 8 at 56, and n = 11 at 70 days follow-up. Increasing mRNA *gdf15* expression correlates with deteriorating cardiac function (**C**,**E**), while plasma GDF15 levels do not significantly correlate with deterioration of cardiac function (**D**,**F**). Dots indicate individual data points across all panels. In panels (**C**–**F**), the solid line represents the best-fit line, derived from linear regression analysis. Mean ± SD. Abbreviations, ESV; End Systolic Volume, GLS; Global Longitudinal Strain. A *p*-value < 0.05 is considered statistically significant.

To better understand the role of GDF15 in developing cardiac dysfunction, we first aimed to determine circulating levels and cardiac tissue expression of Gdf15 in a mouse model of non-ischemic heart failure. During pressure overload-induced HF, the cardiac gene expression of Gdf15 progressively increased over time, as shown by qPCR analysis (Figure 1A). Circulating levels of GDF15 measured in plasma already increased after 3 days post-TAC and remained nonprogressively elevated over time (Figure 1B). As cardiac function worsened over time, as shown by an increase in end systolic volume (ESV), the gene expression of Gdf15 in the heart also increased (*p* < 0.001) (Figure 1C), along with a trend toward higher circulating GDF15 protein levels (*p* = 0.066) (Figure 1D). In line, cardiac expression of Gdf15 correlated to decreased myocardial deformation measured by the global longitudinal strain (GLS) (*p* < 0.001) (Figure 1E), although no association was found between GLS and circulating GDF15 protein levels (*p* = 0.197) (Figure 1F). These results, consistent with observations in patients, suggest that circulating GDF15 levels reflect the presence of cardiac disease in mice.

### 2.2. Reduced Cardiac Function and Adverse Cardiac Remodelling Are More Severe in Gdf15^-/-^ Mice in Response to Cardiac Pressure Overload

**Figure 2:**
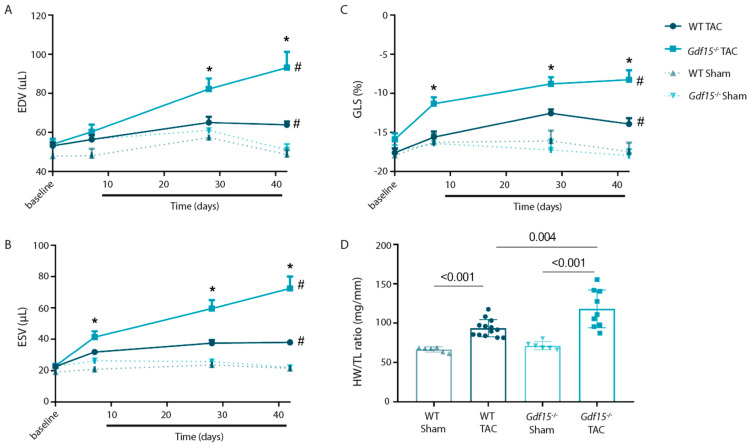
Adverse cardiac function in *Gdf15*^-/-^ mice. Following TAC, both EDV (**A**) and ESV (**B**) increase in WT mice. This increase is aggravated in *Gdf15*^-/-^ mice. GLS is already affected in *Gdf15*^-/-^ mice 1 week after TAC (**C**). Also, the increase in heart weight/ tibia length (HW/TL) ratio is more pronounced in *Gdf15*^-/-^ mice compared to WT (**D**). WT sham n = 6, WT TAC n = 13, *Gdf15*^-/-^ sham n = 6, *Gdf15*^-/-^ TAC n = 9. Mean ± SD. * *p* < 0.05 compared to baseline, # *p* < 0.001 compared to sham controls.

**Figure A1:**
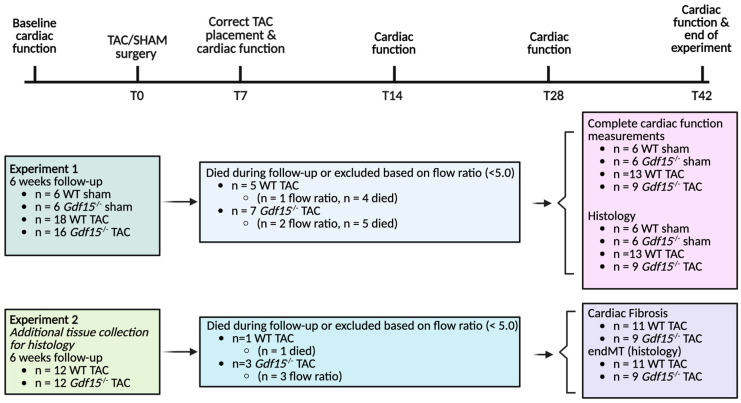
Experimental design and flow chart of *Gdf15*^-/-^ mice experiments. Flow chart of mice experiments 1 and 2 showing mice included at baseline, mice sacrificed after 6 weeks and mice lost during follow-up or excluded based on flow ratios. Five *Gdf15*^-/-^ mice and 4 WT mice in experiment 1 and 1 *WT* mouse in experiment 2 died because of disease development following TAC placement. Two *Gdf15*^-/-^ mice and 1 WT mouse in experiment 1, and 3 *Gdf15*^-/-^ mice in experiment 2 were excluded based on improper placement of the TAC (flow ratio < 5).

**Figure A2:**
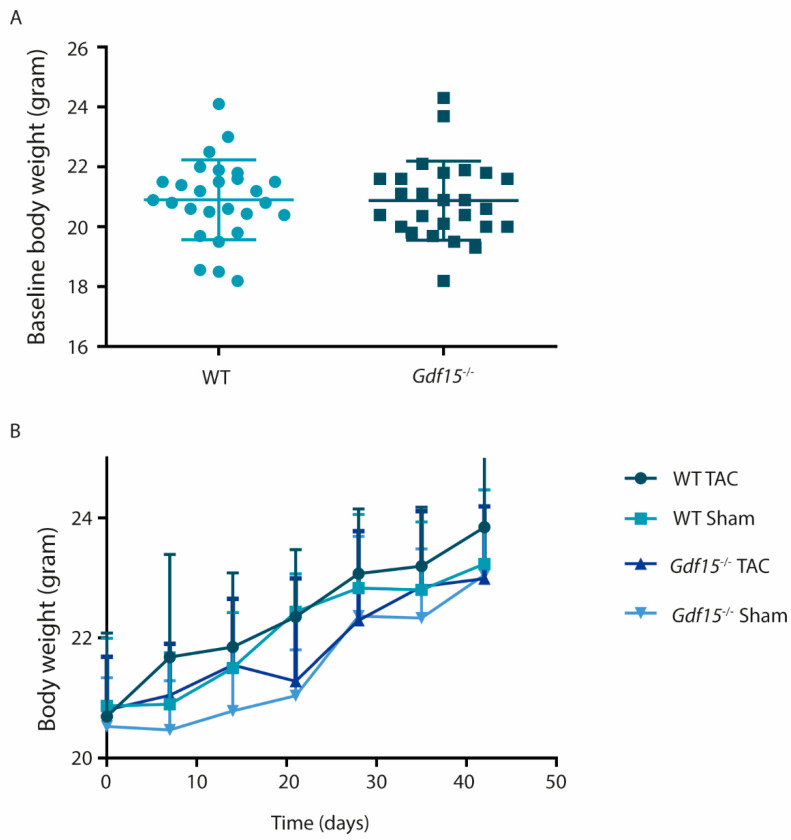
Body weight at baseline and over time post-surgery. At baseline, no differences in body weight of *Gdf15*^-/-^ and their WT littermates could be observed (**A**) n = 27 WT, n = 26 *Gdf15*^-/-^. Over time post-TAC or Sham surgery, no differences in weight gain or loss could be observed between the *Gdf15*^-/-^ mice and the WT littermates (**B**) WT TAC n = 13, WT Sham n = 6, *Gdf15*^-/-^ TAC n = 9, *Gdf15*^-/-^ Sham n = 6.

To assess the functional contribution of GDF15 to cardiac remodelling and HF, *Gdf15*^-/-^ mice and their WT littermates were subjected to pressure overload-induced HF by means of a TAC model. During the 42-day follow-up after TAC, 5 out of 16 *Gdf15*^-/-^ and 4 out of 18 WT mice died due to HF (Figure A1). Body weight was not different between *Gdf15*^-/-^ and WT with or without TAC over time (Figure A2). *Gdf15*^-/-^ mice showed a more severe adverse remodelling of the heart compared to WT littermates after one week of TAC, as seen by an increase in ESV (41.4 ± 10.6 µL vs. 31.2 ± 5.7 µL, *p* = 0.031) and by a decrease in cardiac deformation, reflected by GLS analyses (−11.8 ± 2.8% vs. −15.5 ± 2.7%, *p* < 0.001) (Figure 2B,C) which progressively continued up to 42 days of follow-up. After 42 days, both EDV and ESV were significantly larger in *Gdf15*^-/-^ mice compared to the WT littermates (EDV: 89.1 ± 24.6 µL vs. 64.5 ± 6.6 µL, *p* < 0.001; ESV: 67.2 ± 25.8 µL vs. 39.1 ± 4.9 µL, *p* < 0.001) (Figure 2A,B). Similarly, heart weight/tibia length ratios were increased more in *Gdf15*^-/-^ mice compared to WT mice (118.1 ± 24.1 mg/cm vs. 93.5 ± 11.0 mg/cm, *p* = 0.004) (Figure 2D). In summary, cardiac function is severely impaired in *Gdf15*^-/-^ mice compared to WT littermates, suggesting that differential cardiac remodelling takes place in *Gdf15*-deficient mice in response to cardiac pressure overload.

### 2.3. Genetic Ablation of Gdf15 Causes Structural Changes in Cardiac Tissue Post-TAC

**Figure 3:**
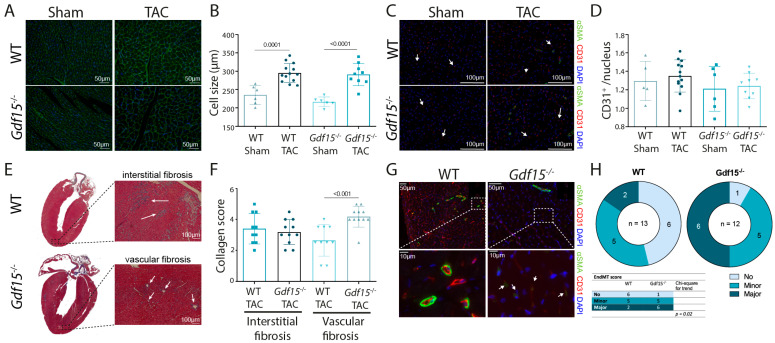
Adverse cardiac remodelling in *Gdf15*^-/-^ mice. Representative pictures of the Wheat Germ Agglutinin (WGA) staining displaying cardiomyocyte cell size (**A**). Cardiomyocyte lining is depicted in green; cell nuclei are depicted in blue. Quantification of cardiomyocyte cell size post-TAC compared to Sham animals in both WT and *Gdf15*^-/-^ (**B**). Post-TAC, both WT and *Gdf15*^-/-^ mice show increased cell size compared to Sham mice. WT Sham n = 6, WT TAC n = 13, *Gdf15*^-/-^ Sham n = 6, *Gdf15*^-/-^ TAC n = 9. Representative CD31 staining (white arrows point at microvessels) in both WT and *Gdf15*^-/-^ post-TAC (**C**). CD31 staining is depicted in red, αSMA in green, and nuclei in blue. Quantification of the CD31 staining per nucleus showed no differences between the 4 groups of animals (**D**). WT Sham n = 6, WT TAC n = 13, *Gdf15*^-/-^ Sham n = 6, *Gdf15*^-/-^ TAC n = 9. Masson’s trichrome collagen staining, with collagen stained in blue (representative pictures shown, white arrows point at collagen deposition) (**E**). Quantification showed no differences in interstitial fibrosis but an increase in vascular fibrosis in the *Gdf15*^-/-^ mice (**F**), WT TAC n = 10, *Gdf15*^-/-^ TAC n = 11. Co-localization of endothelial and mesenchymal markers. (**G**) Representative images of the CD31 (endothelial marker in red) and αSMA (mesenchymal marker in green) staining in both WT and *Gdf15*^-/-^ mice ((**G**), arrows indicate co-localization). CD31 staining is depicted in red, αSMA in green, and nuclei in blue. Semi-quantitative assessment of the co-localization of CD31 and αSMA was more pronounced in *Gdf15*^-/-,^ indicative of EndMT, as shown by a higher proportion of double-positive co-localisation ((**H**), *p* = 0.03). WT TAC n = 13, *Gdf15*^-/-^ TAC n = 12. Mean ± SD. *p* < 0.05 is considered significant. Abbreviations: ESV, End Systolic Volume; EDV, End Diastolic Volume; GLS, Global Longitudinal Strain. WT; Wild type. *Gdf15*^-/-^; *Gdf15* knock-out.

**Figure A3:**
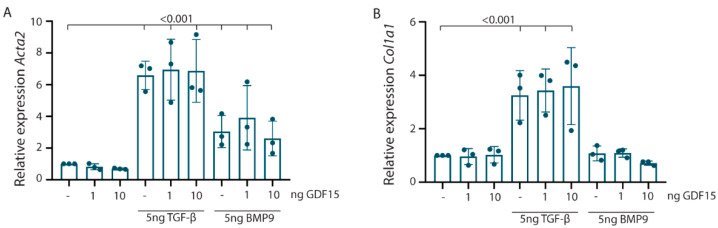
GDF15 does not affect TGFβ or BMP activation of hFCFs. qPCR analysis on expression levels of *Acta2* (**A**) and *Col1a1* (**B**) in hFCFs stimulated with GDF15 in combination with and without 5 ng TGF-β3 or 5 ng BMP9. n = 3 experiments (3 technical replicates each). Mean ± SD. Abbreviations, BMP; Bone morphogenetic protein, Col1a1; Collagen 1a subunit 1, TGF-β; Transforming growth factor-β, TNF; Tissue necrosis factor, αSMA; α-Smooth muscle actin.

To assess structural differences in cardiac remodelling, cardiomyocyte hypertrophy, vessel density, and collagen content were evaluated in cardiac tissue. As expected, cardiomyocytes hypertrophy was increased upon TAC compared with sham-operated mice (235.5 ± 25.9 µm^2^ vs. 295.7 ± 25.3 µm^2^ in WT Littermates (*p* = 0.001) and 216.6 ± 13.0 µm^2^ vs. 291.5 ± 30.3 µm^2^ in *Gdf15*^-/-^ mice (*p* < 0.001)), with no apparent differences between groups in sham mice (235.5 ± 25.9 µm^2^ in WT vs. 216.6 ± 13.0 µm^2^ in *Gdf15*^-/-^, *p* = 0.3) or mice subjected to TAC surgery (295.7 ± 25.3 µm^2^ in WT vs. 291.5 ± 30.3 µm^2^ in *Gdf15*^-/-^, *p* = 1.0) (Figure 3A,B). The number of microvessels per cardiomyocyte was quantified based on CD31 staining (Figure 3C). There was no increase in the number of CD31-expressing microvessels per cardiomyocyte after TAC, nor did we observe a difference between WT mice (1.3 ± 0.2 CD31^+^/nucleus vs. 1.4 ± 0.2 CD31^+^/nucleus, *p* = 0.1) and *Gdf15*^-/-^ mice (1.2 ± 0.2 CD31^+^/nucleus vs. 1.2 ± 0.1 CD31^+^/nucleus, *p* = 0.8) (Figure 3D). It has previously been shown that cardiac interstitial fibrosis in mice increases after TAC [31], and circulating GDF15 levels have been correlated with the degree of fibrosis in human HF patients [32]. We found no significant difference in interstitial collagen content between WT and *Gdf15*^-/-^ mice (WT: 3.4 ± 1.0 vs. Gdf15^-/-^: 3.2 ± 0.9, (arbitrary units, *p* = 0.6)) upon quantification of hearts stained for Mason Trichome (Figure 3E,F). Exogenous addition of recombinant GDF15 had no effect on ACTA2 (encoding α-smooth muscle actin) and COL1A1 (Collagen 1 alpha 1) gene expression in human cardiac fibroblasts in vitro, alone or in combination with TGF-β or BMP-9 (Figure A3). Strikingly, perivascular collagen content was significantly higher in *Gdf15*^-/-^ mice compared to WT littermates (WT: 2.7 ± 1.0 vs. *Gdf15*^-/-^: 4.2 ± 0.7, *p* = 0.0007) (Figure 3E,F). EndMT has been associated with cardiac and perivascular fibrosis [30,33,34]. We determined the degree of co-localization of endothelial and mesenchymal markers (defined as no, minor, or major), which is considered indicative of EndMT [35]. Of the WT mice, 46% of the cardiac endothelial cells had no signs of co-localization, 39% showed minor co-localization, and 15% showed major co-localization, while in *Gdf15*^-/-^ mice, only 8% had no signs of EndMT-like endothelial changes, 42% showed minor, and 50% major signs of EndMT-like endothelial changes (Figure 3G,H, chi-square *p* for trend = 0.02). Our results indicate that progressive HF and adverse cardiac remodelling may be correlated with perivascular fibrosis and suggest EndMT as an underlying mechanism for cardiovascular remodelling in *Gdf15*^-/-^ mice.

### 2.4. Genetic Loss of GDF15 Enhances Activin-Induced Signalling in Endothelial Cells

**Figure 4:**
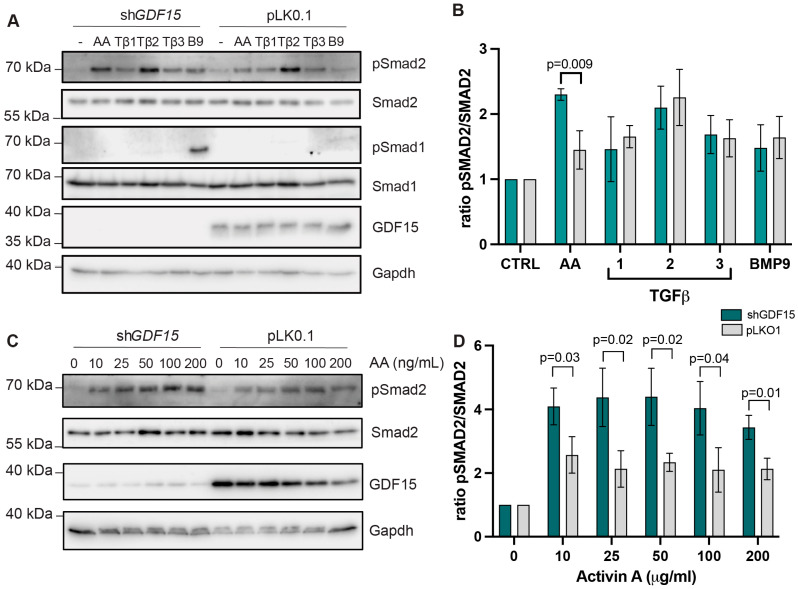
Expression of EndMT markers in Activin A, TGFβ, and BMP9-stimulated control and GDF15 knockdown cells. Knockdown of GDF15 by using a short hairpin RNA (shGDF15) successfully reduced GDF15 protein expression compared to control cells (pLKO.1) (**A**). Activin A stimulation increases Smad2 phosphorylation upon GDF15 knockdown, while Smad1 phosphorylation is unaffected. TGFβ and BMP9 signalling of SMAD proteins is not affected upon knockdown of GDF15 (**A**,**B**). Stimulation with increasing AA concentration results in a dose-dependent increase in Smad2 phosphorylation in both shGDF15 and control cells, whereas GDF15 protein expression decreases in the pLKO.1 control cells upon Activin A (**C**,**D**). Furthermore, Activin A (AA) stimulation increases Smad2 phosphorylation upon GDF15 knockdown, while Smad1 phosphorylation is unaffected. TGF-β and BMP9 signalling of SMAD proteins is not affected upon knockdown of GDF15. Abbreviations: AA; Activin A, Tβ; Transforming growth factor β, B9; Bone Morphogenetic protein 9, GDF15; Growth differentiation factor 15. pLKO1; empty control vector, shGDF15; short hairpin GDF15. Grey bars represent pLKO.1 control cells, while green bars indicate shGDF15 cells. Mean ± SD. n = 3 Western blots. *p* < 0.05 is considered significant.

**Figure A4:**
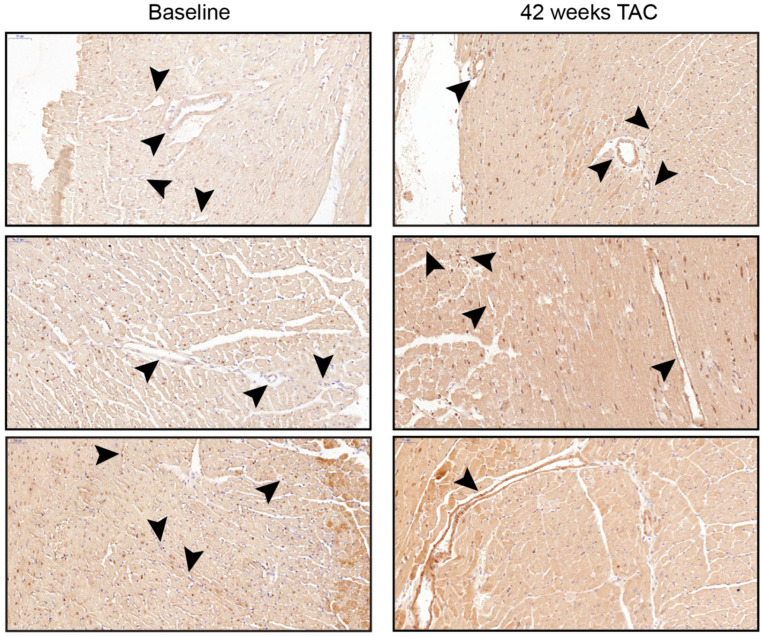
GDF15 is expressed in murine myocardial endothelial cells. Expression is most pronounced in endothelial cells and expression is increased upon TAC. Arrows illustrate GDF-15 expressing endothelial cells. GDF15 is depicted in brown, nuclei in blue.

Given that TGF-β signalling is considered a master regulator of EndMT and loss of TGF-β SMAD2/3 activation has been shown to prevent cardiac fibrosis [30], we aimed to investigate whether responses to TGF-β family ligands were altered upon loss of GDF15. Consistent with previous findings [36], we confirmed that endothelial cells express GDF15, particularly upon TAC, as assessed by immunostaining (Figure A4). Therefore, we first generated stable GDF15-knockdown human umbilical vein endothelial cells (HUVECs) using lentiviral-mediated shRNA transduction. As can be appreciated (Figure 4A), GDF15 is expressed in HUVECs transduced with a control lentiviral vector, and it is effectively downregulated upon GDF15 shRNA expression. Notably, upon stimulation with different TGF-β ligands, including TGF-β1, TGF-β2, TGF-β3, Activin A, and BMP9, GDF15-deficient HUVECs exhibited enhanced Smad2 phosphorylation upon Activin A stimulation compared to control cells (Figure 4A,B, and Table A1), whereas other ligands induced comparable Smad responses in both cell types. Moreover, downregulation of GDF15 led to increased pSmad2 activation in HUVECs at all Activin A doses tested (10–200 ng/mL) (Figure 4C,D and Table A2). These results suggest that loss of GDF15 alters Activin A-induced Smad2 activation in endothelial cells, potentially driving EndMT.

### 2.5. Genetic Loss of GDF15 Leads to Activin-Induced EndMT

**Figure 5:**
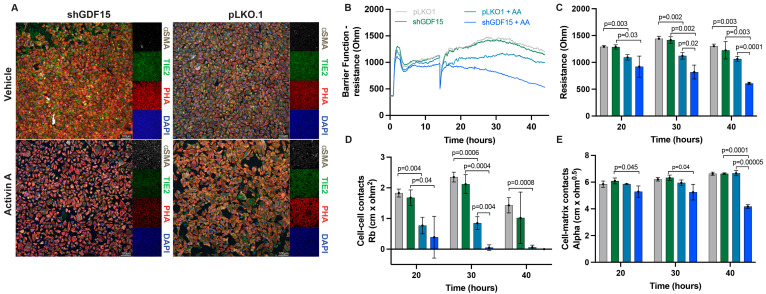
Endothelial barrier integrity is breached in the absence of GDF15 and upon Activin A-induced inflammation. Representative pictures of an EndMT staining of control and GDF15 knockdown endothelial cells. The EndMT staining consists of 4 markers, DAPI as nuclear stain, Tie2 as vascular endothelial marker, αSMA as mesenchymal marker and Phalloidin (PHA) as marker for cellular shape (**A**). Endothelial barrier function is plotted over time for the control condition (pLKO1) and the GDF15-deficient cells (shGDF15), which show a decrease in resistance after AA stimulation, indicating a loss of barrier function. (**B**,**C**). Compared to the control, the lack of GDF15 further decreases the barrier function over time, as seen by a further reduced resistance (*p* < 0.001). Starting after 20 h of stimulation, AA reduces the barrier function for both control and GDF15-deficient cells. After 30 h of AA stimulation, the GDF15-deficient cells have a reduction in barrier function compared to the control cells stimulated with AA (**B**,**C**). A reduced barrier function as consequence of endothelial dysfunction results in a reduced cell–matrix and cell–cell interaction, a reduced cell–cell contact is present after induction of AA for both control and GDF15-deficient cells (**D**), and a reduced cell–matrix interaction is shown to be present for the GDF15-deficient cells after stimulation with AA compared to the GDF15-deficient cells without AA stimulation (*p* < 0.001) (**E**). n = 3 experiments (containing 3 technical replicates each). Grey bars represent pLKO.1 control cells, green bars show shGDF15 cells, teal bars depict pLKO.1 control cells stimulated with AA, and blue bars indicate shGDF15 cells stimulated with AA. Mean ± SD. *p* < 0.05 is considered significant.

Finally, to determine whether Activin A effectively induces endothelial dysfunction and EndMT in the absence of GDF15, we analysed the expression of endothelial and fibroblast-specific markers in control and GDF15 knocked-down HUVECs (Figure 5A). Interestingly, upon stimulation with recombinant Activin A, shGDF15 HUVECs showed irregular expression of the endothelial marker Tie2 and a loss of cobblestone morphology in Phalloidin-stained cells (Figure 5A and Table A3). This suggests that in the absence of GDF15, endothelial cells are more prone to initiate EndMT. During EndMT, endothelial cells acquire a spindle-shaped migratory phenotype, compromising their cell–cell and cell–matrix interactions [37]. Therefore, we used real-time impedance spectroscopy to monitor barrier function in control and shGDF15 HUVECs in response to Activin A (Figure 5B). Over time, Activin A significantly reduces the endothelial barrier function in both control (PLKO1, 1301.93 ± 38.69 vs. 1064.93 ± 48.19, *p* < 0.003) and shGDF15 (1225.93 ± 161.34 vs. 609.17 ± 21.66, *p* < 0.003). However, shGDF15 endothelial cells have a more severe loss of barrier function compared to the control cells after 40 h (1064.93 ± 48.19 vs. 609.17 ± 21.66, *p* < 0.001) (Figure 5B,C). In response to Activin A, a substantial reduction in cell–cell contacts is seen between GDF15 KD cells and control cells post Activin A stimulation (0.05 ± 0.09 vs. 0.85 ± 0.21, *p* < 0.001) at 30 h (Figure 5D). Furthermore, a significant decrease in cell–matrix interactions was observed specifically upon 40 h of Activin stimulation in GDF15-deficient cells compared to the control (4.18 ± 0.14 vs. 6.67 ± 0.19, *p* < 0.001) (Figure 5E). These results suggest that genetic depletion of GDF15 compromises endothelial function, possibly through EndMT, and this effect is further enhanced in the presence of Activin A.

## 3. Discussion

In patients with HF, high circulating GDF15 levels are associated with disease severity and worse outcomes. However, whether these high levels are cause or consequence remains unclear. In a murine model of non-ischemic pressure overload-induced HF, we showed that reduced cardiac function is associated with increased circulating GDF15 levels. Furthermore, GDF15 expression in cardiac tissue gradually increases as HF progresses. Next, we subjected WT and *Gdf15*^-/-^ mice to pressure overload to induce the development of HF. We found that *Gdf15*^-/-^ mice responded strongly to pressure overload, with severe left ventricular systolic dysfunction and dilatation after 42 days. Different from what was shown during early remodelling in *Gdf15*^-/-^, we could not explain these functional differences based on cardiomyocyte hypertrophy [24]. Although interstitial fibrosis was similar, we did observe that more perivascular fibrosis was present in *Gdf15*^-/-^ mice, indicative of vascular dysfunction. Interestingly, in hearts from *Gdf15*^-/-^ mice, increased co-localization of αSMA and CD31 (PECAM-1) was observed, suggestive of EndMT. This was substantiated by in vitro experiments in GDF15-deficient endothelial cells, showing enhanced Smad2 activation, EndMT, and loss of barrier function in response to a subset of TGF-β family ligands, the Activins [38]. We conclude that GDF15 in cardiac tissue is required to prevent left ventricular systolic dysfunction and dilatation via inflammation-induced endothelial dysfunction.

Clinical studies consistently demonstrate that circulating GDF15 levels increase with heart failure severity and are associated with adverse clinical outcomes. At first glance, this appears contradictory to our experimental findings showing aggravated adverse cardiac remodelling in the absence of GDF15. However, elevated biomarker levels during disease progression do not necessarily imply that the molecule itself exerts detrimental effects. Instead, increased GDF15 expression may represent a compensatory stress-response mechanism that is activated in response to ongoing tissue injury and inflammation. Indeed, GDF15 has been proposed to contribute to tissue tolerance during inflammation-induced dysfunction by promoting cellular adaptation and survival under stress conditions [25]. Inflammatory stimuli induce GDF15 production in multiple cell types, potentially supporting vascular integrity and metabolic adaptation during acute or chronic tissue stress [25]. Within this context, elevated circulating GDF15 levels in patients may primarily reflect the severity of ongoing cardiac stress and vascular dysfunction, while simultaneously representing and endogenous protective response aiming at limiting further inflammation-driven tissue damage and adverse cardiac remodelling. Our experimental findings support this concept, as genetic loss of GDF15 resulted in enhanced vascular remodelling, increased mesenchymal marker acquisition by endothelial cells, and aggravated cardiac dysfunction following pressure overload. Together, these findings support the interpretation that GDF15 acts primarily as a compensatory stress-response mediator during cardiac injury, while elevated circulating levels simultaneously reflect the severity of ongoing cardiovascular stress and tissue remodelling. Thus, elevated GDF15 levels in patients may not necessarily indicate pathogenicity of GDF15 itself, but rather activation of an endogenous protective pathway in response to cardiovascular injury.

Fibrosis is a major determinant of cardiac remodelling [39]. Our results indicate that perivascular fibrosis is enhanced in *Gdf15*^-/-^ mice through endothelial dysfunction and potentially EndMT. It has been suggested that increased perivascular fibrosis in the myocardial microvasculature, and thereby reduced microcoronary blood flow, contributes to non-ischemic HF [40,41]. However, the underlying pathophysiologic mechanisms and the contributions of endothelial and vascular dysfunction to the progression of HF remain debated [40]. GDF15 is known to induce angiogenesis [42], and its secretion upon endothelial senescence is associated with enhanced endothelial cell proliferation and migration, thereby preserving vascular function [43]. In addition, it was recently shown that GDF15 can protect from excessive reactive oxygen species production and diabetes-induced endothelial dysfunction in HUVECs and isolated murine aortas [44]. This is consistent with our in vitro results, which showed reduced endothelial function in the absence of GDF15. We explored the association between the absence of GDF15 and endothelial dysfunction, as EndMT may underlie increased perivascular fibrosis [30]. We validated this hypothesis by demonstrating an increased co-localization of CD31 and aSMA, suggestive of EndMT in vivo in the absence of GDF15. These findings are supported by a case report that shows co-localization of CD31 and αSMA on endothelial cells related to cardiac valvular haemangiomas, suggesting that EndMT may play a role in the development of vascular anomalies [45].

Together, these results suggest an association between GDF15 and endothelial dysfunction-related remodelling. GDF15 has been proposed as a biomarker for heart failure progression; this study does not establish it as a marker of endothelial dysfunction in non-ischemic HF. Instead, it suggests GDF15 may reflect endothelial and vascular stress during adverse cardiac remodelling, which is hard to assess directly in routine HF care. Future studies are needed to explore if circulating GDF15 levels relate to vascular dysfunction measures in heart failure patients. Furthermore, comorbidities present in HF patients do contribute to the GDF15 levels, and as such, it is interesting to explore whether GDF15 is a particularly relevant biomarker for a specific HFpEF phenogroup [46,47,48,49,50,51].

Limitations: Although both male and female animals were included in the expression analyses shown in Figure 1, we focused on female animals in the subsequent experiments due to the clinical relevance of non-ischemic heart failure in women. Consequently, sex-specific mechanisms were not examined, and caution is advised when extrapolating our findings to males. Endothelial and vascular dysfunction were not directly assessed using dedicated in vivo vascular function measurements. Although several techniques are available to evaluate vascular function in mice these analyses require specialised experimental setups and were beyond the primary scope of the current study. Instead, endothelial dysfunction was inferred from histological vascular alterations and complementary in vitro endothelial assays. Future studies incorporating direct in vivo vascular function measurements would further strengthen mechanistic insight into the role of GDF15 in cardiovascular remodelling. The in vitro HUVEC model used might not completely represent cardiac endothelium. Validating our results in models using microvascular endothelial cells would further strengthen mechanistic interpretation and could enhance the translational significance of our findings. Another limitation is the lack of an endothelial lineage tracing mouse model that provides insight into the rise in perivascular endothelial-derived mesenchymal cells in the absence of GDF15. However, our study provides strong indications for such a relation as GDF15 is part of the TGF-β family, which are known mediators of chronic inflammation-induced fibrosis, including Activin A.

Our findings provide valuable insights into GDF15’s role in the development of HF. In our studies, we identified GDF15 as an important protector of vascular function by preventing EndMT-like endothelial changes. As mentioned earlier, particularly in HFpEF, it has been postulated that a systemic pro-inflammatory state induces microvascular endothelial dysfunction resulting in excessive perivascular fibrosis and HF [28], though the contribution of EndMT and endothelial dysfunction to HFpEF and non-ischemic cardiac remodelling is debated. Further unravelling the underlying signalling cascade in cardiac tissue inflammation is needed to understand the specific contribution of GDF15 in this process. This will not only provide further insight into a more specific potential of GDF15 as a clinical biomarker but also shed light on potential intervention strategies. From the obesity field, we know that GDF15 is a candidate therapeutic target via the GDF15/GFRAL axis [52]; furthermore, others have shown that pharmacological GDF15 administration had a beneficial effect on food intake in mice [53]. This potential intervention strategy aims at high levels of GDF15 with a strong metabolic effect. As a potential strategy in HF patients, we suggest a more subtle approach to specifically interact with the GDF15 axis to preserve or restore endothelial function.

## 4. Materials and Methods

### 4.1. Animals

To analyse circulating GDF15 levels during the development of HF, a mixed population of male and female C57BL/6J mice (age 9–12 weeks, weight 20–30 g) was used, originally obtained from the Jackson Laboratory and subsequently bred in our own facility. To investigate the causal role of GDF15 in HF, and the increased prevalence of HF with non-ischemic origin in women, we used female *Gdf15*^-/-^ (*Gdf15* KO, kindly provided by Professor Lee, University of Connecticut School of Medicine [54]) and their wild type (C57Bl/6J background) littermates (age 11–13 weeks, weight 18–24 g). All mice received standard chow and water ad libitum and were housed in filter-top cages under a 12/12 light/dark cycle. The researcher who performed surgery on block-randomly assigned animals was blinded to animal groups (the blocking factor was day of surgery). Technicians and observers, who were blinded to animal groups, performed the respective operations, data acquisition, and analyses. All surgeries were performed in a dedicated mouse operating room. All animal experiments were approved by the Ethical Committee on Animal Experimentation of the University Medical Centre Utrecht (Utrecht, The Netherlands) and conform to the ‘Guide for the care and use of laboratory animals’. Transverse aortic constriction (TAC) surgeries were performed in a dedicated mouse operating room by an experienced surgeon, as previously reported [55,56].

### 4.2. TAC Procedure

The TAC procedure was performed as described previously [57]. Anaesthesia was induced by intraperitoneal (i.p.) injection of medetomidine hydrochloride (1.0 g/kg body weight), midazolam (Dormicum^®^, Roche, 10.0 mg/kg), and fentanyl (Janssen-Cilag, 0.1 mg/kg). Mice were intubated and connected to a respirator with a 1:1 oxygen–air ratio (175 strokes/minute, 250 µL stroke volume). A core body temperature of 37 °C was maintained during surgery by continuous rectal temperature monitoring and an automatic heating blanket. To place the transverse aortic constriction, the aortic arch was reached between two ribs after a midline incision in the anterior neck. The TAC was placed between the brachiocephalic artery and the left common artery against a blunt 27-gauge needle with a 7-0 suture, followed by prompt removal of the needle. The surgical wounds were closed, and subcutaneous atipamezole hydrochloride (3.3 mg/kg), flumazenil (0.5 mg/kg), and buprenorphine (0.15 mg/kg) were used as an antagonist and analgesia. One additional subcutaneous injection of buprenorphine (0.15 mg/kg) was administered 12 h later. Sham-operated mice underwent the same procedure without aortic binding. Correct placement of the TAC was confirmed after 1 week by Doppler flow measurements of the carotid arteries (only flow ratios > 5 between the left and right carotids were considered successful and included). To assess GDF15 expression in the heart and levels in plasma during heart failure development, TAC surgery was performed on a mixed population of male and female C57BL/6J mice (n = 117, n = 13 baseline, n = 9–13 for 3, 7, 14, 21, 28, 35, 42, 56, 70 days follow-up). During follow-up, 2 animals were excluded based on flow ratio, and 14 animals died due to disease development. For a 6-week survival assessment, 18 WT and 17 *Gdf15*^-/-^ received TAC surgery, and 6 WT and 6 *Gdf15*^-/-^ were sham operated. Mortality due to disease development after TAC was similar between groups with, 4 WT and 5 *Gdf15*^-/-^ animals dying (*p* = 0.42, Fisher’s exact test). In addition, 1 WT and 2 *Gdf15*^-/-^ were excluded based on flow ratios (indicative of improper TAC placement), and final analysis was performed on 13 WT TAC, 12 *Gdf15*^-/-^ TAC, 6 WT sham, and 6 *Gdf15*^-/-^ sham. Due to technical issues with embedding, some tissues were unsuitable for reliable histological assessment. We therefore included an additional set of 12 WT and 12 *Gdf15*^-/-^ mice exposed to TAC (42 days of follow-up), with the specific aim of supplementing the histological assessment of fibrosis and the presence of EndMT-like endothelial changes. In this cohort of mice, 1 WT mouse died during follow-up, and 3 *Gdf15*^-/-^ mice were excluded based on flow ratio (Figure A1).

### 4.3. Echocardiography

To evaluate cardiac structure and function, echocardiography was performed at baseline and on days 7, 14, 35, and 42 after TAC [57]. During the procedure, mice were anaesthetised with inhalation of 2.0% isoflurane in a mixture of oxygen and air (1:1). Heart rate, respiratory rate, and rectal temperature were continuously monitored, and body temperature was maintained between 36.0 and 38.0 °C using heating lamps. Three-dimensional images were reconstructed from two-dimensional images taken on the short axis of the heart at multiple levels in both end systole and end diastole, with respiratory gating. To analyse global longitudinal strain, Vevo strain speckle-tracking software was used. Image acquisition and analysis were performed with the dedicated Vevo^®^ 2100 System and Software (Fujifilm VisualSonics Inc., Toronto, ON, Canada).

### 4.4. Termination

Mice were exsanguinated after anaesthesia with sodium pentobarbital (60.0 g/kg). Blood was collected via orbital puncture into EDTA-coated tubes for plasma separation [57]. The vascular system was flushed with 5 mL of phosphate-buffered saline (PBS) through right ventricular puncture. Heart weight and tibia length were measured. Half of the heart was fixed in formalin for 24 h and then embedded in paraffin. The other half was snap-frozen in liquid nitrogen for RNA and protein extraction.

### 4.5. Analysis of GDF15 Circulating Levels

To assess the circulating GDF15 levels in progressive heart failure, GDF15 was measured in a TAC model [56,57] characterising cardiac remodelling. In short, 104 mice underwent TAC surgery and were terminated at different time points post-TAC (3, 7, 14, 21, 28, 35, 42, 56, 70 days). Furthermore, 13 mice were terminated at baseline (no surgery). Final analysis was performed on 101 mice (baseline + post-TAC). GDF15 levels are measured with a mouse-specific GDF15 ELISA (MGD150, Quantikine ELISA, R&D Systems Inc., Minneapolis, MN, USA).

### 4.6. Immuno-Histochemical Staining of Murine Tissue and Scoring

Paraffin-embedded murine hearts were cut and prepared for immunofluorescent staining. To examine cell size, Wheat Germ Agglutinin (WGA) staining was performed. To visualise blood vessels, both micro and macro, double immunofluorescent staining for α-Smooth Muscle Actin (αSMA) and Platelet Endothelial Cell Adhesion Molecule-1 (Pecam1, CD31) was performed. Due to technical issues with embedding, some tissues were unsuitable for reliable histological assessment. We therefore included an additional set of 12 WT and 12 *Gdf15*^-/-^ mice exposed to TAC (42 days of follow-up), with the specific aim of supplementing the histological assessment of fibrosis and the presence of EndMT-like endothelial changes. In this cohort of mice, 1 WT mouse died during follow-up, and 3 *Gdf15*^-/-^ mice were excluded based on flow ratio (Figure A1). All scoring procedures were independently performed by two blinded investigators. The average score of both observers was used for the final analysis. In cases of discrepancy between observers, a third blinded investigator was consulted to obtain consensus.

Fibrosis scoring: Quantification of collagen density was performed in five independent myocardial fields per heart section using Masson’s Trichrome staining with semi-quantitative analysis. Sections were scored separately for interstitial and perivascular fibrosis based on the intensity of the trichrome staining (scores 1–5: no, minor, moderate, heavy, severe staining). Interstitial fibrosis was defined as collagen deposition localised between cardiomyocytes within the myocardial interstitium, whereas perivascular fibrosis was defined as collagen deposition surrounding vascular structures. Scores were averaged per field and subsequently averaged between the two blinded observers for final analysis.

Endothelial/mesenchymal marker co-localization analysis: For analysis of endothelial/mesenchymal marker co-localization, five independent myocardial fields per heart were evaluated. Only small vascular structures were included in the analysis. Larger vessels with a clearly distinguishable CD31-positive endothelial lining and αSMA-positive vascular smooth muscle layer were excluded to prevent physiological vascular smooth muscle staining from influencing the analysis.

Co-localization was defined as the simultaneous expression of CD31 and αSMA within the same cell. The degree of co-localization per field was semi-quantitatively scored (scores 1–3: no, minor, major co-localization). Scores were averaged per field and subsequently averaged between the two blinded observers for final analysis. Detailed information on the scoring is provided in Table 1.

To detect GDF15 in mouse sections, we performed DAB-based immunohistochemistry as previously described [36]. Representative photomicrographs were taken with a Leitz Diaplan system and a Nikon DXM1200 digital camera (Nikon Instruments, Melville, NY, USA) or a Leica DMIL LED microscope with 10× magnification, as chosen by two independent observers in discussion. All antibodies used are listed in Table 2.

### 4.7. Primary Endothelial Cell Isolation and Culture

Human umbilical vein endothelial cells (HUVECs) were isolated as described elsewhere [57,58] and maintained in EGM-2 (Lonza, Basel, Switzerland) supplemented with 10% FBS and 0.1% penicillin/streptomycin (Invitrogen, Breda, The Netherlands). All experiments were performed with cells grown to near confluency between passages 6 and 8. Isolation and culture of primary cells were approved by the ethics committee of the Leiden University Medical Centre, the Netherlands. This is in accordance with the principles outlined in the Declaration of Helsinki.

### 4.8. In Vitro EndMT Assays

HUVECs were seeded at confluence in 12-well plates (for Western blot assays) or Lab-Tek II chamber slides (for immunofluorescence assays) in EBM2 complete medium containing 10% FBS. Next day, the cells were stimulated with TGFβ1 (1 ng/mL), TGFβ2 (1 ng/mL), TGFβ3 (1 ng/mL), Activin A (50 ng/mL) or BMP9 (1 ng/mL) (R&D systems, Abingdon, UK) for 72 h in EBM2 medium without FBS, and endothelial and mesenchymal specific markers were analysed by immunofluorescent labelling and/or Western blotting using the protocols described below.

### 4.9. Endothelial Barrier Function Assays

Endothelial barrier function was analysed using impedance-based cell monitoring with an electric cell–substrate impedance sensing system (ECIS Zθ, Applied Biophysics, Troy, NY, USA). HUVECs were seeded onto 1% gelatine-coated ECIS arrays, each containing 8 wells with 10 gold electrodes (2 per well; 8W10E PET, Applied Biophysics). The cell seeding density was estimated to be ~50,000 cells/cm^2^. After stabilisation of the chip in growth medium, HUVECs were seeded for at least 16 h in complete EBM2 growth medium containing 10% FBS. Then, the medium was replaced by EBM2 growth medium without FBS, supplemented with either recombinant Activin A (50 ng/mL) (R&D Systems, Abingdon, UK) or control vehicle (ligand buffer: 4 mM HCl, 0.1% BSA). Stabilisation of the barrier was monitored in real time over 40 h. The multiple frequency/time (MFT) mode was used to assess the barrier and monolayer confluence in real time. Mathematical modelling of cell–matrix contacts (alpha) and cell–cell interactions (Rb) was performed as previously described [59].

### 4.10. Immunofluorescent Labelling of Cultured Cells

After culture in EndMT medium (see above), cells were fixed with 4% formaldehyde for 30 min at RT, washed with glycine for 5 min, permeabilized with 0.2% Triton X-100, and blocked in PBS containing 5% BSA for 1 h. The cells were incubated overnight at 4 °C with the primary antibody in the blocking solution with gentle shaking. The next day, the cells were washed five times in washing buffer (PBS containing 0.05% Tween-20 and 1% BSA) and incubated with secondary antibodies (Alexa Fluor 488 goat anti-mouse IgG and Alexa Fluor 555 anti-rabbit IgG) or phalloidin in PBS containing 0.5% BSA for 1 h. Finally, the cells were washed five times in washing buffer and mounted in Prolong Gold containing DAPI (Invitrogen). Preparations were imaged in a Leica SP5 confocal scanning laser microscope. The antibodies used are listed in Table 1.

### 4.11. Western Blotting

Cells were seeded into 12-well plates and cultured until they reached confluence. Then, cells were stimulated as indicated and washed with cold PBS. Lysates were prepared using 2× Sample Buffer (containing 10% SDS, 62.5 mM Tris-HCl pH 6.8, 0.002% Bromophenol Blue, 0.7135 M (5%) β-mercaptoethanol, 10% glycerol) and boiled for 5 min before loading. Electrophoresis was performed in 10% SDS/polyacrylamide gels. Proteins were transferred onto nitrocellulose membranes (at 0.5 A and 100 V in ice for 1 h). Blots were blocked overnight at 4 °C in Tris-Buffered Saline with 0.1% Tween 20 and 5% non-fat dry milk. Immunodetection was carried out using primary antibodies (see Table 3), followed by horseradish peroxidase-conjugated secondary antibodies (anti-mouse, anti-rabbit from GE Healthcare, Eindhoven, Netherlands, or anti-Rat from R&D Systems, Abingdon, UK). Detection employed an ECL system (Fisher Scientific, Landsmeer, The Netherlands) and a ChemiDoc documentation system (Bio-Rad, Hercules, CA, USA). The antibodies are listed in the table below. Band intensity was measured to quantify expression, which was normalised to GAPDH levels. Non-treated cells served as controls, and protein expression was normalised to GAPDH, with ratios calculated relative to CTRL-treated cells.

### 4.12. Lentivirus Production

Lentiviral vectors were produced in HEK293T cells as described previously [61]. Lentiviral vectors expressing specific shRNAs were purchased from Sigma-Aldrich, Darmstadt, Germany (MISSION^®^ shRNA). Human GDF15 (NM_004864) coding sequence was amplified by PCR from 1 ug of RNA from human umbilical vein endothelial cells (HUVECs) using the oligos hGDF15EcoRV-FW: ATAGGATATCATGCCCGGGCAAGAACTCAG and hGDF15XhoI-RV: CTATCTCGAGTCATATGCAGTGGCAGTCTT. Amplified DNA was subsequently digested with EcoRV and XhoI and ligated into a previously described lentiviral pLV-IRES-Puro vector. The nucleotide sequence of the obtained pLV-GDF15 vector was confirmed by Sanger sequencing. Stable ECFCs infected with either control (empty) viruses or pLV-GDF15 were selected with puromycin (1 µg/mL) for 48 h two days after infection. The shRNA constructs used in this study are shown in Table 4.

### 4.13. Statistical Analysis

We used end systolic volume (ESV) as a clinical surrogate for HF as primary outcome measure to perform group size calculation. We powered based on 2D echo imaging for a difference of 0.4 mm ESV, with a power of 90%, alpha of 0.05, a standard deviation of 0.29 mm^2^ based on historical data from our group, and a maximum mortality of 20%, resulting in 15 animals per group for TAC. Data are presented as mean ± SD. Mixed models were used for repeated measurements (end diastolic volume (EDV), ESV, and global longitudinal strain (GLS)), with a random intercept for each mouse and as fixed factors group and time point. To determine whether the time course of the parameters differed across groups, the interaction between group and time point of measurement was also included in the model. Heart weight/tibia length, Wheat Germ Agglutinin Staining (WGA), Masson’s trichrome, and CD31 were analysed with a Student’s *t*-test, scores for EndMT were analysed using the Chi-square test for trend. Statistical analyses were performed using SPSS version 21 and GraphPad Prism 6 and *p* ≤ 0.05 was considered statistically significant. All in vitro results are shown as mean ± SD, and representative data are shown in the figures.

## 5. Conclusions

In the current study, we gain deeper mechanistic insight into the role of GDF15 in cardiac size and function within a pressure overload HF model. We demonstrate that the absence of GDF15 leads to the development of severe HF, primarily due to endothelial dysfunction, EndMT-like endothelial changes, and perivascular fibrosis formation. Our findings suggest that GDF15 provides protective effects on cardiac homeostasis, especially by supporting endothelial cell function. Finally, the idea that GDF15 levels might directly indicate vascular dysfunction warrants more research into GDF15’s role in protecting endothelial cells.

## Figures and Tables

**Table 1:** Semi-quantitative scoring system for fibrosis and EndMT co-localization.

Score	Interstitial Fibrosis	Perivascular Fibrosis	Score	EndMT Co-Localization
1: No	absence of fibrosis	absence of fibrosis	1: No	
2: Minor	sparse and focal collagen deposition with minimal disruption of the normal myocardial architecture	visible separation between the vessel wall and surrounding myocardial tissue without clear histological evidence of fibrosis	2: Minor	occasional co-localised cells within the field
3: Moderate	multifocal areas of collagen deposition with extension into the surrounding myocardial tissue	fibrosis surrounding the vessel without infiltration into the adjacent myocardial tissue	3: Major	numerous co-localised cells present throughout the field
4: Heavy	extensive collagen-rich regions with marked infiltration throughout the myocardium and clear disruption of cardiomyocyte organisation	moderate fibrosis with limited extension into the surrounding tissue		
5: Severe	widespread, dense, and highly interconnected fibrotic regions causing severe disruption of myocardial tissue architecture and cardiomyocyte connectivity	extensive fibrosis with clear infiltration into the surrounding myocardium		

**Table 2:** Antibodies used for immunohistochemistry.

Staining Mice	Antibody	Dilution	Provider	Reference
	WGA-FITC conjugate	1:40	Sigma-Aldrich, Saint Louis, MO, USA	L4895
	αSMA-FITC	1:400	Sigma-Aldrich, Saint Louis, MO, USA	F3777
	PECAM-1	1:1500	Santa Cruz, Dallas, TX, USA.	Sc-1506
	Polyclonal anti-rabbit biotin	1:200	Vector laboratories, Newark, CA, USA	BA1000
	Streptavidin-Alexa 555	1:1000	Invitrogen, ThermoFisher scientific, Eugene, OR, USA	S21381
	Masson’s Trichrome	n.a.	Sigma-Aldrich, Saint Louis, MO, USA	HT15
	GDF15	1:2000	Abcam, Cambridge, UK	Ab39999
	Streptavidin−biotin complex		DAKO, Agilent, Santa Clara, CA, USA	
Staining cells	Antibody	Dilution	Provider	Reference
	Alpha-SMA	1:2000	Abcam, Cambridge, UK	Ab124964
	Tie2	1:100	Abcam, Cambridge, UK	Ab24859
	FN1	1:400	Sigma-Aldrich, Saint Louis, MO, USA	F7387
	Phalloidin	1:100	ThermoFisher scientific, Eugene, OR, USA	
	Anti-mouse-IgG-Alexa Fluor^TM^ 488	1:200	Invitrogen, ThermoFisher scientific, Eugene, OR, USA	
	Anti-Rabbit IgG, Alexa Fluor^TM^ 555	1:200	Invitrogen, Invitrogen, ThermoFisher scientific, Eugene, OR, USA	

**Table 3:** Antibodies used for Western blot.

Antibody to	Application	Dilution	Supplier	Reference
GDF15	WB, IHC,	1:1000 (WB), 1:2000 (IHC)	Abcam (Cambridge, UK)	Ab39999
p-Smad1/5	WB	1:1000	Cell Signalling Technology (Leiden, The Netherlands)	#9511
p-Smad2	WB	1:1000	Homemade	Nakao et al. EMBO. 1997;16(17):5353-62 [60]
Gapdh (6C5)	WB	1:10,000	Millipore (Burlington, MA, USA)	CB1001
Smad1	WB	1:1000	Cell Signalling Technology	#6944
Smad2	WB	1:1000	BD (Franklin Lakes, NJ, USA)	610842

**Table 4:** shRNA constructs.

Gene	Reference (MISSION shRNA—Sigma-Aldrich)	Species
*GDF15*	TRCN0000058388, TRCN0000058391. GDF15 targeting viruses were prepared by pooling two different viral stocks (1:1)	Human
pLK0.1 (Empty vector control)	SHC-001	N/A

## Data Availability

The data produced and/or examined during this study can be obtained from the corresponding author upon reasonable request.

## Abbreviations

The following abbreviations are used in this manuscript:

αSMA	α-Smooth Muscle Actin
BMP	bone morphogenetic proteins
EDV	End Diastolic Volume
EndMT	Endothelial-to-Mesenchymal transition
ESV	End Systolic Volume
GDF15	Growth differentiation factor 15
GLS	global longitudinal strain
HF	Heart Failure
HFpEF	Heart Failure with preserved Ejection Fraction
HUVEC	Human umbilical vein endothelial cell
PBS	phosphate-buffered saline
PECAM	Platelet endothelial cell adhesion molecule
TAC	Transverse Aortic Constriction
TGF	transforming growth factor
WGA	Wheat Germ Agglutinin
WT	Wild Type

## Appendix A

**Table A1:** Ratio of phosphorylated SMAD2 expression in shGDF15 cells versus control pLKO.1 cells.

	Ratio pSMAD1/SMAD1			Ratio pSMAD2/SMAD2		
	shGDF15	pLKO.1	*p*-Value	shGDF15	pLKO.1	*p*-Value
**AA**	n.d.	n.d.		2.3 ± 0.08	1.45 ± 0.29	0.009
**TGFβ1**	n.d.	n.d.		1.46 ± 0.50	1.65 ± 0.17	0.6
**TGFβ2**	n.d.	n.d.		2.10 ± 0.33	2.26 ± 0.43	0.6
**TGFβ3**	n.d.	n.d.		1.69 ± 0.30	1.63 ± 0.28	0.8
**BMP9**	2,177,486.2 ± 860,579.5	1,798,599.3 ± 918,595.2	0.6	1.48 ± 0.35	1.64 ± 0.32	0.6

Stable GDF15 knocked-down human umbilical vein endothelial cells and control cells were stimulated with Activin A (AA), TGFβ1, TGFβ2, TGFβ-3, and Bone Morphogenic Protein 9 (BMP). Protein expression of (p)SMAD-1 and (p)SMAD was assessed by Western blot. Protein expression in shGDF15 cells was compared with expression in control pLKO-1 cells. phosphoSMAD-1 is undetectable in all conditions except for BMP9 stimulation, where expression is not different between shGDF15 and pLKO.1 cells. The ratio of pSMAD2/SMAD2 is significantly higher in shGDF15 cells compared pLKO.1 cells for all treatment conditions.

**Table A2:** Ratio of pSMAD2/SMAD2 Protein expression in Activin A stimulated shGDF15 vs. control cells.

	Ratio pSMAD2/SMAD2		
AA (ng/mL)	shGDF15	pLKO.1	*p*-Value
**10**	4.09 ± 0.58	2.57 ± 0.57	0.03
**25**	4.38 ± 0.92	2.13 ± 0.57	0.02
**50**	4.40 ± 0.90	2.34 ± 0.28	0.02
**100**	4.04 ± 0.84	2.10 ± 0.70	0.04
**200**	3.43 ± 0.38	2.13 ± 0.34	0.01

Stable GDF15 knocked-down human umbilical vein endothelial cells and control cells were stimulated with increasing concentrations of Activin A (AA). Protein expression of (p)SMAD2 was assessed by Western blot. Expression was quantified by measuring band intensity and expression normalised for GAPDH expression. Non-treated cells served as controls and protein expression was normalised for GAPDH and the ratio was calculated compared to CTRL-treated cells. Protein expression in shGDF15 cells was compared with expression in control pLKO-1 cells. The ratio of pSMAD2/SMAD2 is significantly higher in shGDF15 cells compared pLKO.1 cells for all AA concentrations.

**Table A3:** Protein expression of different EndMT markers in stimulated shGDF15 vs. control cells.

	VWF			FSP-1			αSMA		
	shGDF15	pLKO.1	*p*-Value	shGDF15	pLKO.1	*p*-Value	shGDF15	pLKO.1	*p*-Value
**AA**	0.96 ± 0.53	1.06 ± 0.65	0.85	1.11 ± 0.65	1.03 ± 0.59	0.88	0.95 ± 0.35	0.97 ± 0.52	0.96
**TGFβ1**	1.44 ± 0.33	0.64 ± 0.16	0.02	0.87 ± 0.07	0.76 ± 0.09	0.17	1.10 ± 0.08	0.95 ± 0.14	0.18
**TGFβ2**	1.30 ± 0.42	0.92 ± 0.23	0.25	0.77 ± 0.19	0.42 ± 0.23	0.11	0.89 ± 0.03	1.37 ± 0.32	0.06
**TGFβ3**	1.78 ± 0.23	0.82 ± 0.15	0.004	0.48 ± 0.21	1.02 ± 0.03	0.01	1.13 ± 0.36	1.01 ± 0.14	0.61
**BMP9**	1.22 ± 0.51	0.62 ± 0.21	0.13	0.64 ± 0.13	0.36 ± 0.14	0.07	0.93 ± 0.15	0.94 ± 0.30	0.96

Stable GDF15 knocked-down human umbilical vein endothelial cells and control cells were stimulated with Activin A (AA), TGFβ1, TGFβ2, TGFβ-3, and Bone Morphogenic Protein 9 (BMP). Protein expression of von Willebrand Factor (VWF), fibroblast-specific protein (FSP-1) and alpha Smooth Muscle Acting (αSMA) was assessed by Western blot. Expression was quantified by measuring band intensity and expression normalised for GAPDH expression. Non-treated cells served as controls and protein expression was normalised for GAPDH and the ratio was calculated compared to CTRL-treated cells. shGDF-15 cells display increased VWF expression upon stimulation with TGFβ1 and TGFβ3, while FSP-1 expression is decreased upon TGFβ3 stimulation. Expression of αSMA is not different between shGDF14 and pLKO.1 control cells.
